# An Evaluation of the Different Serum Markers Associated with Mortality in Crimean–Congo Hemorrhagic Fever

**DOI:** 10.5041/RMMJ.10393

**Published:** 2020-10-14

**Authors:** Yusuf Kenan Tekin, Aynur Engin

**Affiliations:** 1 Department of Emergency Medicine, Sivas Cumhuriyet University Medical Faculty, Sivas, Turkey; 2 Department of Infectious Diseases and Clinical Microbiology, Sivas Cumhuriyet University Medical Faculty, Sivas, Turkey

**Keywords:** Crimean-Congo hemorrhagic fever patient, emergency department, mean platelet volume-to-platelet count ratio, red cell distribution width-to-platelet count ratio

## Abstract

**Background:**

Crimean–Congo hemorrhagic fever (CCHF) is a tick-borne viral disease with a high mortality rate. Although CCHF has been widely investigated over the past decade, a review of the literature indicated no data on the prognostic capacity of the mean platelet volume-to-platelet count ratio (MPVPCR) and the red cell distribution width-to-platelet count ratio (RDWPCR) for the systemic inflammatory response in patients with CCHF. This study aimed to evaluate the prognostic ability of MPVPCR and RDWPCR on mortality in patients with CCHF.

**Methods:**

A total of 807 patients that were admitted to the Cumhuriyet University Hospital’s Emergency Department from January 2010 to December 2018 were involved. The RDWPCR and MPVPCR were separately calculated via absolute blood red cell and platelet counts at the time of admission. Before performing receiver-operating characteristic (ROC) curve analysis to define the optimum cut-off values of MPVPCR and RDWPCR stepwise logistic regression analysis was used to determine the predictive factors related to mortality in CCHF patients.

**Results:**

Values of both MPVPCR and RDWPCR were significantly lower in survivors than in non-survivors (MPVPCR: 0.20±0.23 versus 0.55±0.55, *P*<0.001; RDWPCR: 0.27±0.32 versus 0.77±0.77, *P*<0.001, respectively). The MPVPCR (odds ratio [OR], 5.95; *P*=0.048) was an independent predictor for the prognosis of mortality in CCHF patients. The area under the curve in the ROC curve analysis for MPVPCR was 0.876 with a cut-off of 0.21 (sensitivity 87%, specificity 76%).

**Conclusion:**

At the time of admission, MPVPCR might be a useful predictor of mortality in patients with CCHF.

## INTRODUCTION

Crimean–Congo hemorrhagic fever (CCHF) is a tick-borne viral disease with a high mortality rate; it has been reported in many parts of the world including Africa, Eastern Europe, the Middle East, and Asia.^1^ The CCHF mortality rate varies between 5% and 30%, and it has been reported to be 5.4% in Turkey.^2^,^3^

The clinical signs of the disease manifest themselves in both non-hemorrhagic and hemorrhagic stages. Early symptoms of CCHF, such as headache, myalgia, back pain, fever, and weakness, occur in the first stage. The second-stage symptoms usually appear four days after disease onset and include hemorrhage and bleeding from the nose, gums, lungs, skin of the hands and legs, stomach, and intestines. A combination of thrombocytopenia and leukopenia can be observed, especially at this stage. The majority of deaths occur between 5 and 14 days after disease onset due to excessive bleeding, persistent fever, anemia, circulatory shock, and disseminated intravascular coagulation.^4^

Early identification of the clinical course of the disease enables appropriate monitoring, supportive measures, and appropriate patient treatment. Therefore, we need a rapid laboratory marker for the accurate prediction of disease outcome.

Recently, both the mean platelet volume-to-platelet count ratio (MPVPCR) and the red cell distribution width-to-platelet count ratio (RDWPCR) have been studied as novel and rapid laboratory markers to predict mortality in various conditions or diseases. Previous studies revealed the role of RDWPCR as a useful inflammatory index in some diseases such as systemic lupus erythematosus, chronic hepatitis, acute pancreatitis, and severe burn injury.^5^–^8^ Other studies have also shown that MPVPCR can be a predictor marker for mortality in patients with severe sepsis.^9^

Although CCHF has been widely investigated over the past decade, a review of the literature indicated no data on the prognostic capacity of MPVPCR and RDWPCR for the systemic inflammatory response in patients with CCHF. Hence, this study aimed to evaluate MPVPCR and RDWPCR in the prognosis of mortality in patients with CCHF.

## MATERIALS AND METHODS

A retrospective record-based study was performed on CCHF patients admitted to the Emergency Department of Cumhuriyet University Hospital between January 2010 and June 2018. The hospital is located in a CCHF high-risk region, where the disease is endemic, and serves as a tertiary care reference hospital for the northeastern part of Turkey. In total 834 CCHF cases that were confirmed by a reverse transcriptase-polymerase chain reaction and viral-RNA detection were included in the study.

The exclusion criteria were as follows: patients taking antithrombotic or anticoagulant medications that can affect platelet count and volume; patients with a hematologic disease, cancer, liver disease, heart or renal failure, chronic infectious disease, vascular disease, or rheumatologic disease. After applying these criteria, 27 patients were excluded from the study.

Ethical approval for this study was obtained from the Ethics Committee of the Cumhuriyet University, protocol number 2019-04/55; the study was conducted in accordance with the principles of the Declaration of Helsinki.

Demographic and medical variables including age, sex, and symptoms onset were obtained at the time of admission. Data related to blood cell counts including white blood cell (WBC), hemoglobin (Hb), platelet counts, mean platelet volume (MPV), neutrophils, lymphocytes, red cell distribution width (RDW), and high-sensitivity C-reactive protein (hs-CRP) were extracted from first lab test results in the patients’ electronic medical records.

The MPVPCR and RDWPCR were calculated as:

MPVPCR=absolute MPV/mean platelet count

and

RDWPCR=RDW/mean platelet count

Statistical analysis was conducted with the SPSS software version 22.0 for Windows (IBM Corp., New York, NY, USA). Parametric or non-parametric tests were performed to analyze data according to normal or abnormal distribution, respectively. Continuous variables are presented as mean±standard deviation (SD), and categorical variables as numbers (percentages). Continuous variables were compared with the Student *t* test, and categorical variables with the chi-square test or the Fisher exact test, as appropriate, to assess the significance of intergroup differences. Stepwise logistic regression analysis was used to determine the predictive factors for mortality in patients with CCHF. Receiver-operating characteristic (ROC) curve analysis was also performed to examine the sensitivity and specificity of MPVPCR and RDWPCR on mortality in patients with CCHF. A *P* value <0.05 was considered statistically significant.

## RESULTS

A total of 807 CCHF patients with a sex distribution of 332 women (41.1%; mean age 49.2±1.7 years) and 475 men (58.9%; mean age 47.7±1.8 years) were recruited in this study. As shown in Table 1, no statistically significant difference was observed between the two groups of survivors and non-survivors in terms of age, gender, and mean symptom duration. However, and not surprisingly, the complications development level was found to be more frequent in non-survivors than in survivors (98.0% versus 3.2%, *P*<0.001) (Table 1).

**Table 1:** Demographic and Clinical Features of the Survivors and Non-survivors of Crimean-Congo Hemorrhagic Fever.

Feature	Survivors (*n*=758, %)	Non-Survivors (*n*=49, %)	*P* Value (Student’s *t* test)
Age, years (mean±SD)	48.0±17.1	53.5±18.8	0.052

Gender			
Male	442 (58.3)	33 (67.3)	0.124*
Female	316 (41.7)	16 (32.7)	

Mean duration of symptoms, days (mean±SD)	5.0±0.7	5.2±0.7	0.126

Complications development			
Yes	24 (3.2)	48 (98.0)	<0.001†
No	734 (96.8)	1 (2.0)	

* Chi-square test.

† Fisher’s exact test.

Table 2 presents the most common complications found among non-survivors, i.e. somnolence (71.4%), mucosal hemorrhage (63.3%), petechiae (57.1%), and acute renal failure (34.7%).

**Table 2:** The Most Common Symptoms and Complications Found among Non-survivors (*n*=49).

Complication	*n* *	%
Somnolence	35	71.4
Mucosal hemorrhage	31	63.3
Petechiae	28	57.1
Acute renal failure	17	34.7
Others †	6	12.2

* Some of the non-survivors had more than one complication.

† Including subarachnoid hemorrhage, alveolar hemorrhage, and hemothorax.

From Table 3, it is noteworthy that the WBC, neutrophil, lymphocyte, hs-CRP, MPV, RDW, MPVPCR, and RDWPCR levels were found to be statistically lower in survivors than in non-survivors. As expected, the platelet level was found to be very low in non-survivors as compared to survivors (41.4±35.9 versus 84.2±46.7, *P*<0.001).

**Table 3:** The Mean Values of Blood Parameters of Survivors versus Non-survivors.

Variables	Survivors(*n*=758, mean±SD)	Non-survivors(*n*=49, mean±SD)	*P* Value(Student’s *t* test)
White blood cells (10^3^/μL)	4.59±3.85	6.94±4.72	<0.001*
Hemoglobin (g/dL)	13.93±2.06	14.61±2.43	0.060
Platelets (10^3^/μL)	84.19±46.73	41.43±35.91	<0.001*
Neutrophils (10^3^/μL)	1.91±1.50	5.25±3.81	<0.001*
Lymphocytes (10^3^/μL)	0.66±0.44	1.00±0.73	<0.001*
High-sensitivity C-reactive protein (mg/L)	13.47±19.64	61.53±41.41	<0.001*
Mean platelet volume (fL)	9.87±1.21	10.27±1.17	0.027*
Red cell distribution width (%)	13.33±1.19	13.89±2.12	0.003*
Mean platelet volume-to-platelet count ratio	0.20±0.23	0.55±0.55	<0.001*
Red cell distribution width-to-platelet count ratio	0.27±0.32	0.77±0.77	<0.001*

* Indicates significance

Logistic regression analysis showed that neutrophil (odds ratio [OR]=0.15; *P<*0.001), lymphocyte (OR=0.32; *P<*0.001), hs-CRP (OR=3.79; *P=*0.001), and MPVPCR (OR=5.95; *P=*0.048) levels were independently predictive of the mortality risk in CCHF patients (Table 4).

**Table 4:** Mortality-related Variables Inform Logistic Regression Analysis (*n*=807).

Independent Variables	Odds Ratio	95% Confidence Interval	*P* Value
Neutrophils (10^3^/μL)	0.15	0.71–0.31	<0.001
Lymphocytes (10^3^/μL)	0.32	0.15–0.69	0.004
High-sensitivity C-reactive protein (mg/L)	3.79	1.71–8.39	0.001
Red cell distribution width (%)	1.19	0.99–1.43	0.072
Mean platelet volume-to-platelet count ratio	5.95	1.01–34.90	0.048
Red cell distribution width-to-platelet count ratio	1.00	0.17–5.87	0.998

**Figure 1:**
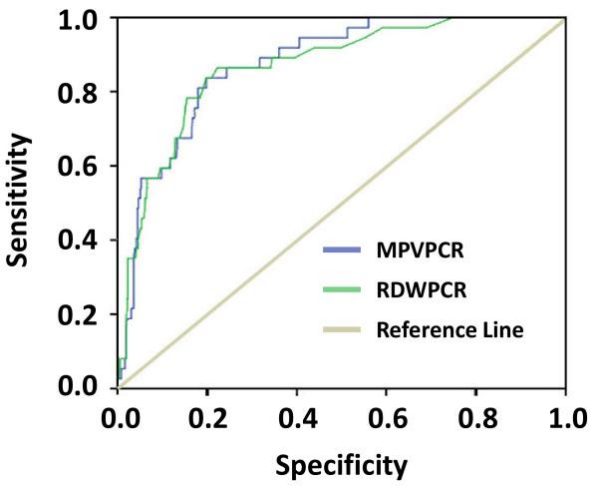
Receiver-operating Characteristic Curve of Red Cell Distribution Width-to-Platelet Count Ratio and Mean Platelet Volume-to-Platelet Count Ratio, for the Prediction of Mortality in Patients with Crimean–Congo Hemorrhagic Fever.

The ROC curve analysis showed that MPVPCR had an area of 0.876 and a cut-off of 0.21, with a sensitivity of 87% and a specificity of 76% (Table 5, Figure 1).

**Table 5:** Cut-off Value, Sensitivity, and Specificity of Red Cell Distribution Width-to-Platelet Count Ratio (RDWPCR) and Mean Platelet Volume-to-Platelet Count Ratio (MPVPCR) for Predicting Mortality in Patients with Crimean–Congo Hemorrhagic Fever.

Value	RDWPCR	MPVPCR
Cut-off value	0.30	0.21
Sensitivity	0.87	0.87
Specificity	0.78	0.76
AUC (95% CI)	0.868 (0.812–0.925)	0.876 (0.828–0.923)

AUC, area under the curve; CI, confidence interval.

The mortality rate of patients with CCHF was also evaluated, and it was found to be 6.1%.

## DISCUSSION

The present study is the largest series of cases of CCHF reported from Turkey. The study revealed that early fatalities in CCHF cases presented with higher neutrophil, lymphocyte, MPVPCR, and hs-CRP levels on admission; hence, these parameters may be early predictors of mortality in CCHF patients. Moreover, the data enable a proposal of MPVPCR cut-off values to estimate the prognosis of mortality in CCHF cases.

Many previous studies have reported the association of hemorrhagic symptoms such as petechiae, ecchymosis, and large purpuric areas with disease severity.^10^,^11^ Furthermore, bleeding from the nose, gums, intestines, and lungs in severe clinical cases, leading to multiple organ failure, has also been observed.^4^,^12^ In addition, somnolence was found to be a predictor of mortality in many other studies.^13^ The clinical findings of this study were similar to those previously reported in other studies.^14^,^15^ The most frequently observed symptoms and complications were noted as somnolence, mucosal hemorrhage, petechiae, and acute renal failure.

The mortality rate was 6.1% in the current study, which is similar to the average rate in other studies performed in Turkey.^16^ Mortality has also been associated with CCHF viral load and older age at presentation.^3^,^17^ Similar to previous studies, a higher mean age in non-survivors was observed in the present study (but this was not statistically significant).

It has been shown that the mean incubation period following a tick bite is between 3 and 7 days.^18^,^19^ Similar to the previous studies, the mean time for onset of symptoms in this study was 5 days.

Although the factors determining CCHF severity have not been clearly revealed, early determination of disease severity is fundamental for disease management aimed at reducing patient mortality. Moreover, there is no definitive treatment methodology for the disease; however, supportive antiviral therapy, applied with blood and blood products, is the cornerstone of treatment options. Hence, developing an easy and reliable marker of disease severity is important.

As observed in this and many other studies, there are a number of hemogram parameters, including WBC, neutrophils, MPV, and platelets, which can indicate the existence and severity of the body’s immune system response, apart from the reported important biochemical parameters in CCHF patients including ferritin, procalcitonin, and CRP.^20^

In recent years, many laboratory blood parameters, including WBC, neutrophils, lymphocytes, hs-CRP, MPV, and RDW, have been studied to predict mortality in various diseases.^3^,^21^,^22^ Moreover, CCHF cases and relationship between WBC, platelets, neutrophils, and MPV, have also been described previously.^23^ Similar to observations reported by the above-mentioned studies,^3^,^21^ non-survivors in the present study presented with a decreased platelet count but with increased WBC, neutrophil, lymphocyte, hs-CRP, MPV, RDW, MPVPCR, and RDWPCR levels.

The relationships of WBC, neutrophils, MPV, and platelets with survival are already known. However, this is the first study to show the possible association of MPVPCR with the progression to mortality in CCHF patients. Although RDWPCR has been indicated as a new laboratory index in predicting mortality in various diseases, this present study did not find it applicable to CCHF patients.^7^,^24^ In the present study, logistic regression analysis showed that neutrophils (OR=0.15; *P*<0.001), lymphocytes (OR= 0.32; *P*<0.001), hs-CRP (OR=3.79; *P*=0.001), and MPVPCR (OR=5.95; *P*=0.048) were independently predictive of the risk of mortality in CCHF patients. Hence, the cut-off of >0.21 for MPVPCR levels may be a useful tool for the prediction of mortality in CCHF disease. Furthermore, the risk of mortality may be 6-fold higher in patients with CCHF whose MPVPCR values exceed the cut-off levels at admission, an important finding of this study, which reveals the potential of MPVPCR values as an independent predictor for mortality in CCHF patients.

## STRENGTH AND LIMITATIONS

This study is the first to show that MPVPCR may be an independent predictor for mortality in CCHF patients. However, some limitations in this current study should be mentioned. Since all the patients with CCHF were recruited from a single center, and all data were obtained from the medical record database, bias in patient selection was unavoidable. Moreover, further prospective studies are needed to confirm the suitability of MPVPCR as a mortality predictor in CCHF due to the retrospective nature of this study and the lack of longitudinal observations.

## CONCLUSION

In conclusion, the present study is the first to reveal the clinical importance of MPVPCR as a potentially useful predictor for mortality in CCHF patients. The study results indicated that MPVPCR was independently associated with CCHF. Since MPVPCR testing is easy, reliable, inexpensive, and widely available, including this test at admission might be a useful predictor for mortality prognosis in CCHF patients. Further studies should be done to validate the role of MPVPCR in the management of CCHF patients.

## Abbreviations

CCHF: Crimean–Congo hemorrhagic fever
Hb: hemoglobin
hs-CRP: high-sensitivity C-reactive protein
MPV: mean platelet volume
MPVPCR: mean platelet volume-to-platelet count ratio
OR: odds ratio
RDWPCR: red cell distribution width-to-platelet count ratio
SD: standard deviation
WBC: white blood cell
